# P-1849. Investigating the Impact of SARS-CoV-2 on the Immune Response to Other Pathogens Using VirScan Epitope Profiling

**DOI:** 10.1093/ofid/ofae631.2010

**Published:** 2025-01-29

**Authors:** Linda M Sircy, Elizabeth M Krantz, Ryan S Basom, Louise E Kimball, Rachel Blazevic, Khaleel Yahya, Terry L Stevens-Ayers, Peter D Han, Robin A Prentice, Lea Starita, Trevor Bedford, Cecile Viboud, Michael J Boeckh, Alpana Waghmare

**Affiliations:** Fred Hutchinson Cancer Center, Seattle, Washington; Fred Hutch Cancer Center, Seattle, Washington; Fred Hutchinson Cancer Center, Seattle, Washington; Fred Hutchinson Cancer Center, Seattle, Washington; Fred Hutchinson Cancer Center, Seattle, Washington; Fred Hutchinson Cancer Center, Seattle, Washington; Fred Hutchinson Cancer Center, Seattle, Washington; University of Washington, Seattle, Washington; University of Washington, Seattle, Washington; University of Washington, Seattle, Washington; Fred Hutchinson Cancer Center, Seattle, Washington; Fogarty International Center, National Institutes of Health, Bethesda, Maryland; Fred Hutchinson Cancer Center, Seattle, Washington; Fred Hutchinson Cancer Center; Seattle Children's Hospital, Seattle, Washington

## Abstract

**Background:**

The emergence and rapid spread of SARS-CoV-2 since 2019 caused substantial global morbidity and mortality and continues to burden healthcare systems worldwide. Public health measures implemented to reduce SARS-CoV-2 spread also reduced circulation of other seasonal respiratory viruses, likely causing gaps in population-level immunity. Whether SARS-CoV-2 infection may dampen immune responses against other pathogens is highly debated and not well understood.

**Figure 1.**
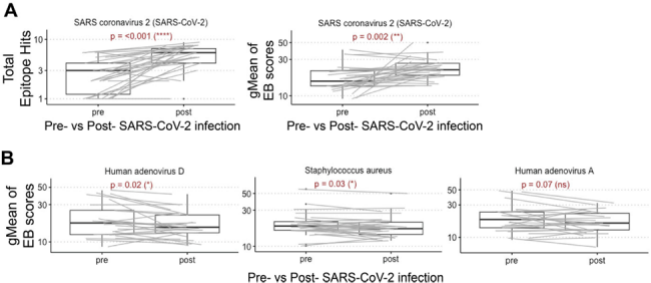
VirScan analyses of serum samples pre- and post- clinically confirmed SARS-CoV-2 infection. (A) VirScan total epitope hits and geometric mean (gMean) of epitope binding (EB) scores for SARS-CoV-2 pre- and post- clinically confirmed SARS-CoV-2 infection. (B) gMean of EB scores for select pathogens pre- and post- clinically confirmed SARS-CoV-2 infection. Timepoints included in the VirScan analyses were between 15-174 days prior to and 31-155 days after clinically confirmed SARS-CoV-2 infection. Gray lines denote paired samples for individual subjects. Statistically significant adjusted p values of < 0.05 were determined using paired t test with Holm correction.

**Methods:**

In 2022, we conducted a longitudinal study of immunocompetent adults ( > 18 years) with recent SARS-CoV-2 infection or vaccination history and increased risk of social or occupational exposure to respiratory viruses. Subjects were followed for 6 months and weekly symptom surveys, blood samples at enrollment, 3 and 6 months post-enrollment, and nasal swabs at report of symptoms were collected. Swabs were tested by RT-PCR (OpenArray) for 27 respiratory pathogens, including SARS-CoV-2. Serum antibodies were assessed by VirScan PhIP-seq for specificity to a broad library of antigen epitopes for clinically relevant pathogens, measuring total epitope hits and epitope binding (EB) scores, a measure similar to antibody titer.

**Results:**

Of 43 subjects with paired serum samples > 1 week prior to and > 28 days after clinically confirmed SARS-CoV-2 infection, 32 subjects with viral load < Ct 22.2 were assessed by VirScan. SARS-CoV-2 epitope hits and EB scores were significantly increased post-infection compared to pre-infection (Fig 1A). SARS-CoV-2 infection was associated with reduced EB scores for adenovirus D and *S. aureus* (Fig 1B). However, SARS-CoV-2 infection was not associated with a statistically significant decline of epitope hits or EB scores for most other clinically relevant pathogens.

**Conclusion:**

We assessed broad changes in the antibody repertoires of subjects prior to and after SARS-CoV-2 infection for clinically relevant pathogens at the epitope level by VirScan. Together these data suggest that SARS-CoV-2 infection is not associated with differential patterns of antibody repertoire kinetics for most non-coronavirus respiratory pathogens in immunocompetent adults. Further analyses in larger cohorts with longer follow-up are needed to confirm these findings.

**Disclosures:**

**Michael J. Boeckh, MD PhD**, Allovir: Advisor/Consultant|Allovir: Grant/Research Support|AstraZeneca: Advisor/Consultant|AstraZeneca: Grant/Research Support|Merck: Advisor/Consultant|Merck: Grant/Research Support|Moderna: Advisor/Consultant|Moderna: Grant/Research Support|Symbio: Advisor/Consultant **Alpana waghmare, MD**, Allovir: Grant/Research Support|Ansun Biopharma: Grant/Research Support|GlaxoKlineSmith: Advisor/Consultant|GlaxoKlineSmith: Grant/Research Support|Pfizer: Grant/Research Support|Vir: Advisor/Consultant

